# Supplementary material for: Erythrocyte Plasma Membrane Lipid Composition Mirrors That of Neurons and Glial Cells in Murine Experimental In Vitro and In Vivo Inflammation
Source: Cells. 2023 Feb 9;12(4):561. doi: 10.3390/cells12040561 (PMC9953778; doi:10.3390/cells12040561)
Supplement: Supplementary file 1 [file cells-12-00561-s001.zip › cells-2175476-supplementary.pdf]

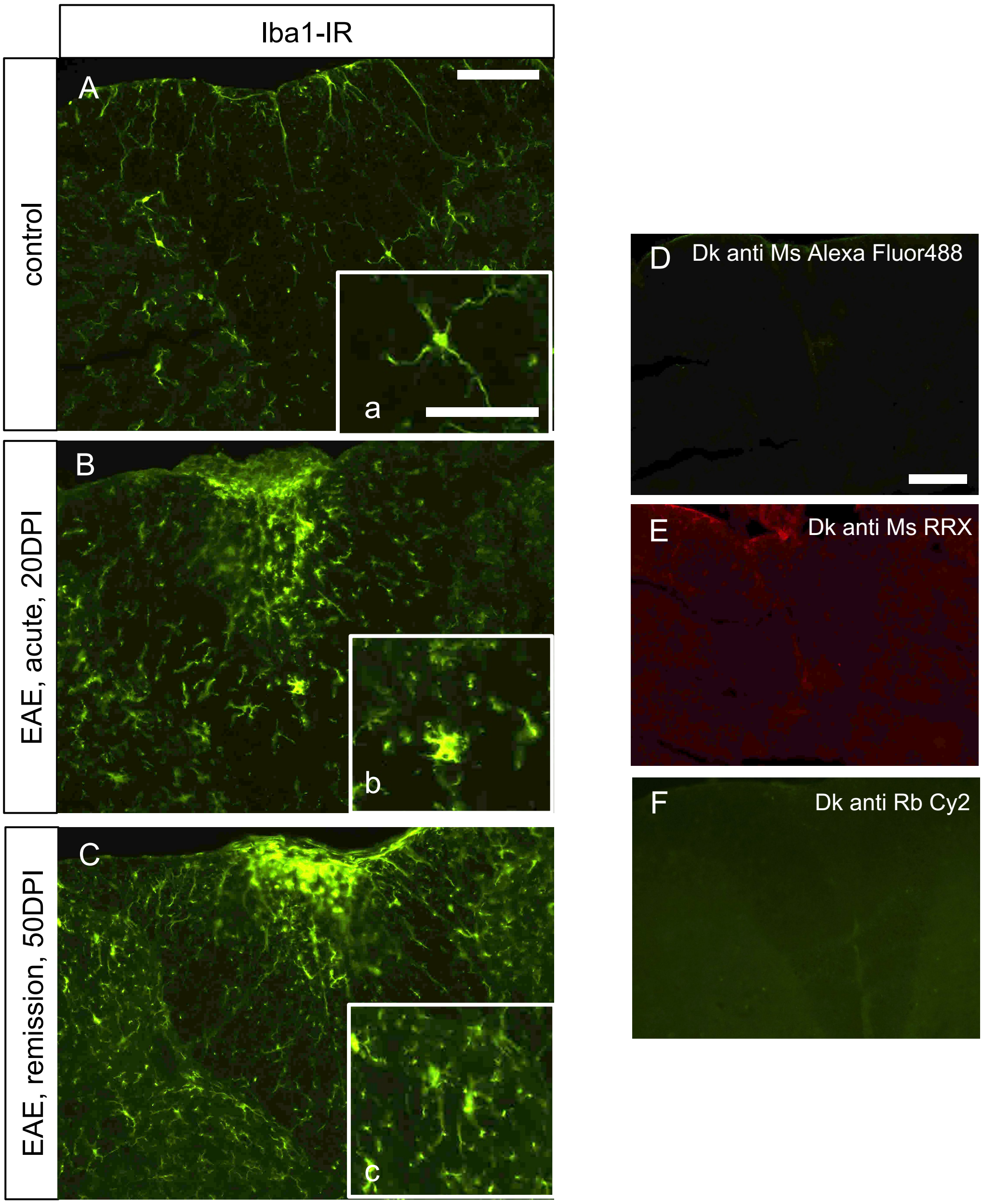


**Figure S1. Iba-1 immunostaining in spinal cord**

A-C: Iba-1 immunostaining in the spinal cord in control mice (A), during acute (B) and remission (C) phases of EAE. The inserts a, b, c illustrate the morphology of Iba-1-positive microglial cells. Scale bar A, B, C: 100 µm; a, b, c: 25 µm.

D-F: immunohistochemical controls using secondary antisera. Scale bar D, E, F: 100 µm

**Table S1.** Fatty acid components obtained from RBC membrane phospholipids of mice at the three different phases of the disease.

| | CTR (n=10) | EAE ( n=7) | REM (n=7) |
|---|---|---|---|
| Fatty acids ( %rel quant.) | mean±SD | mean ±SD | means ±SD |
| 16:0 | 35.13±2.08 | 37.83±1.36** | 37.01±5.21 |
| 6 cis 16:1 | 0.29±0.19 | 0.16±0.08 | 0.24±0.16 |
| 9 cis 16:1 | 0.52±0.23 | 0.38±0.17 | 0.71±0.23# |
| 18:0 | 18.59±4.11 | 21.23±1.91 | 14.90±1.37### § |
| 9 trans 18:1 | 0.06±0.05 | 0.09±0.07 | 0.05±0.02 |
| 9cis 18:1 9 | 13.85±1.66 | 13.45±1.47 | 16.04±1.64##§ |
| 11 cis 18:1 | 2.04±0.28 | 2.13±0.28 | 2.99±0.39### §§ |
| mtrans 18:2 | 0.04±0.03 | 0.06±0.04 | 0.03±0.02 |
| 18:2 omega-6 | 10.05±1.72 | 9.38±0.85 | 10.83±1.49# |
| 18:3 omega-6 | 0.19±0.06 | 0.20±0.06 | 0.04±0.03 §§ |
| 18:3 omega-3 | 0.17±0.09 | 0.07±0.03* | 0.07±0.02 § |
| 20:0 | 0.30±0.13 | 0.40±0.13 | 0.31±0.19 |
| 11 cis 20:1 | 0.36±0.08 | 0.37±0.09 | 0.47±0.27 |
| 20:3 omega-6 DGLA | 2.17±1.07 | 1.91±0.36 | 2.47±0.57# |
| 20:4 omega -6 ARA | 9.76±2.30 | 7.43±0.55* | 8.23±1.83 |
| monotrans 20:4 ARA | 0.08±0.06 | 0.04±0.02 | 0.02±0.02 § |
| 20:3 omega-3 EPA | 0.47±0.22 | 0.29±0.13 | 0.52±0.33 |
| 13 cis 22:1 | 0.05±0.03 | 0.07±0.06 | 0.04±0.03 |
| 22:5 omega-3 DPA | 0.72±0.37 | 0.46±0.10 | 0.61±0.37 |
| 22:6 omega-3 DHA | 5.05±1.48 | 3.16±0.75** | 3.63±1.85 |
| 15cis 24:1 | 0.03±0.04 | 0.00±0.00 | 0.01±0.01 |
| SFA | 54.02±5.59 | 59.46±2.32* | 52.22±4.94## |
| MUFA | 17.13±2.17 | 16.57±1.66 | 20.46±2.35## §§ |
| PUFA | 28.58±6.65 | 22.91±1.81* | 26.39±5.05 |
| omega- 6 | 22.17±4.83 | 18.92±1.26 | 21.57±3.25 |
| omega-3 | 6.41±2.01 | 3.99±0.90** | 4.82±2.29 |
| omega-6/omega-3 | 3.63±0.72 | 4.74±1.21* | 4.47±1.48 |
| SFA/MUFA | 3.18±0.43 | 3.59±0.43 | 2.58±0.34### §§ |
| ARA/EPA | 23.33±6.51 | 28.93±10.02 | 20.87±11.12 |
| ARA/DHA | 1.98±0.30 | 2.45±0.51* | 2.57±0.91 |
| omega-3/PUFA*100 | 22.42±3.34 | 17.30±3.16** | 17.62±5.29§ |
| tot trans | 0.17±0.09 | 0.19±0.10 | 0.11±0.05 |

[1]Fatty acids are obtained as FAME[1] (fatty acid methyl esters) after work-up as described in the Experimental part. Values are reported as relative quantitative percentages of each FAME, recognized and calibrated with standard references in the GC analysis. The values are expressed as mean± sd of n replicates. Statistical analyses were performed using Graph Pad 6. Unpaired t-test*: EAE vs CTR ; *: pvalue ≤ 0.045; ** pvalue ≤ 0.0097; #: REM vs EAE : #p value: 0.048; ##0.009; ###0.0005 §:REM vs CTR : § p value: 0.04; §§: 0.008; §§§ ≤ 0.0001

| FAME | diet |
|---|---|
| 14:0 | 0.4 |
| 16:0 | 14.6 |
| 9 cis 16:1 | 0.5 |
| 18:0 | 2.6 |
| 9 cis 18:1 | 21.2 |
| 11 cis 18:1 | 1.3 |
| 18:2 omega-6 | 49.6 |
| 18:3 omega-3 | 5.6 |
| 20:0 | 0.4 |
| 11cis 20:1 | 0.5 |
| 20:4 omega-6 ARA | 0.2 |
| 20:5 omega-3 EPA | 0.9 |
| 22:6 omega-3 DHA | 2.1 |
| omega-6/omega-3 | 5.1 |
| omega-3/PUFA | 14.7 |

**Table S2.**
Fatty acid composition of the diet

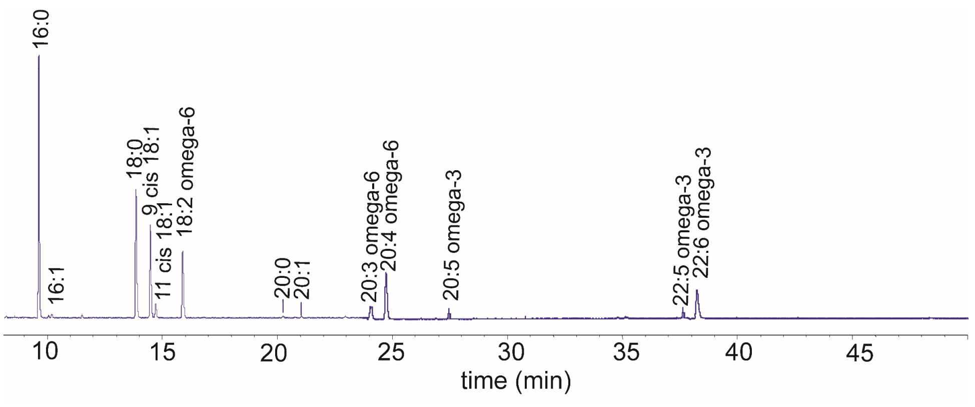


**Figure S2.** Representative gas chromatographic (GC) run and FAME (fatty acid methyl ester) obtained from RBC membrane lipidome and recognized by standard references as described in the Experimental Part.

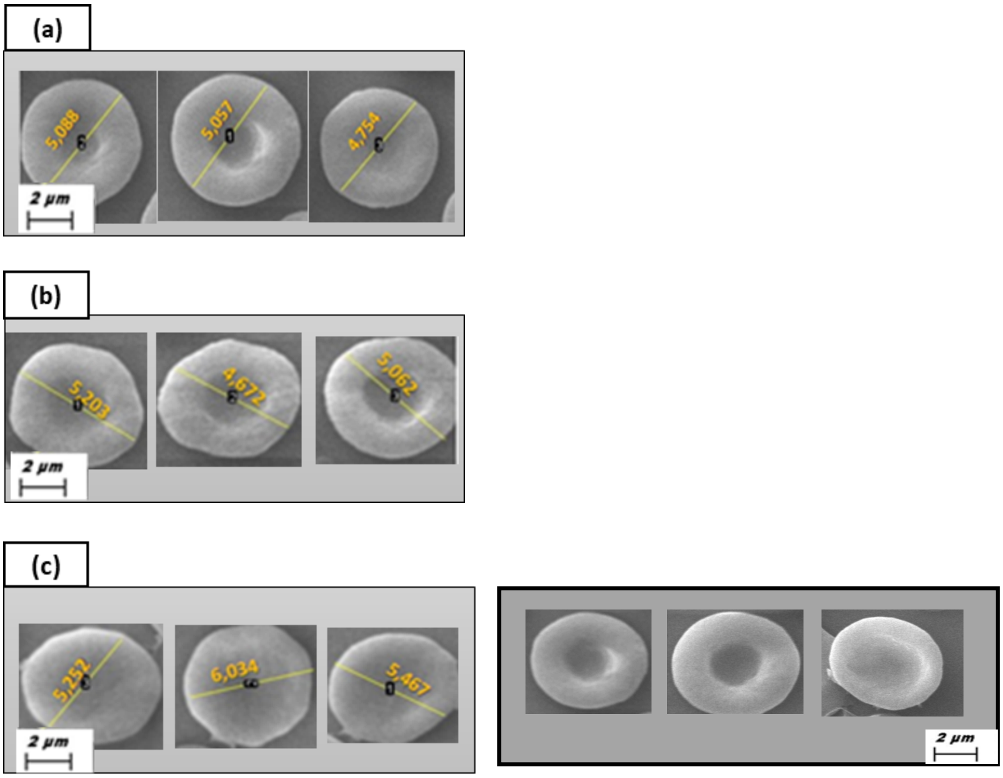


**Figure S3. SEM images of RBCs.**
SEM images of RBCs derived from control (a) and experimental animals in acute (b) and recovery (c) phases of EAE.

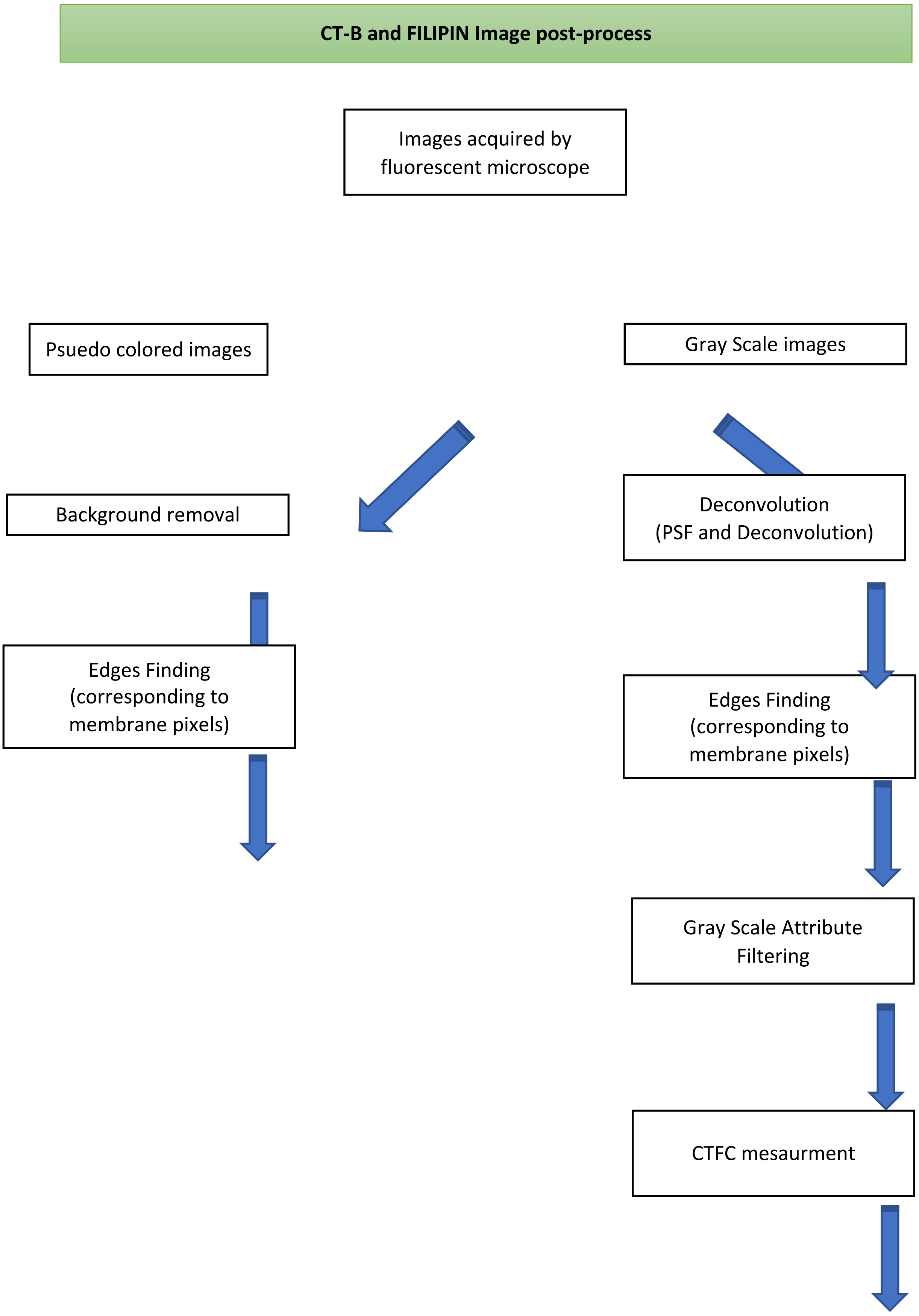


**Figure S4.** Post-processing image analysis of CT-B and Filipin in RBCs (see methods for further details)

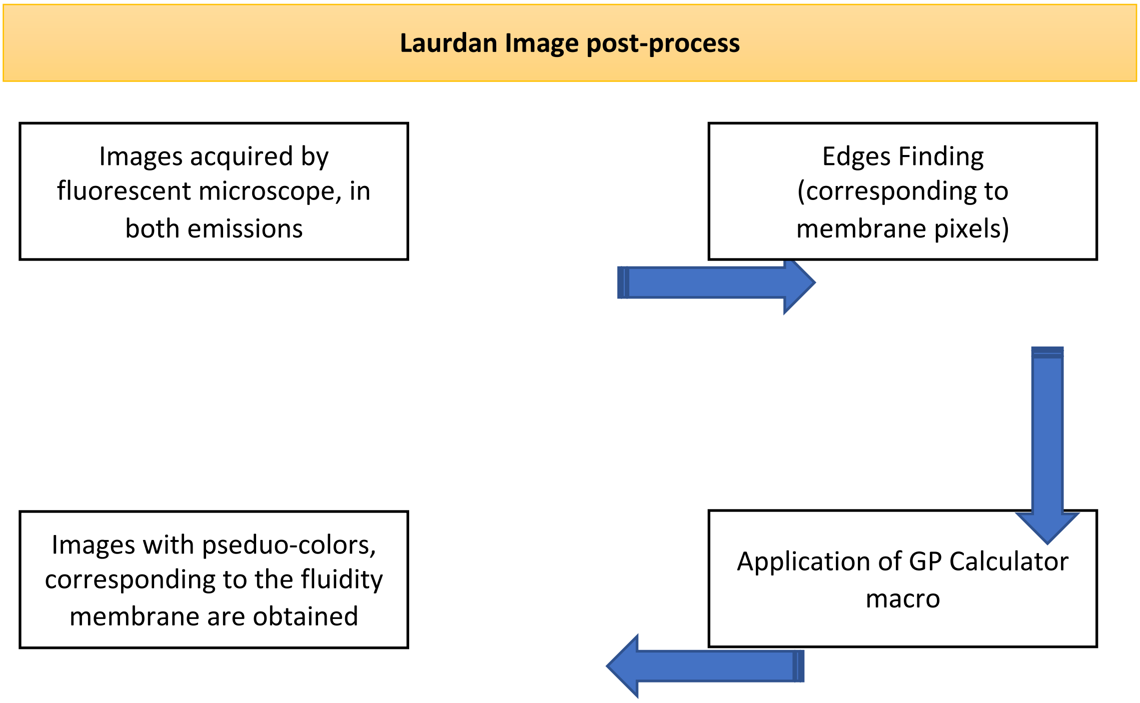


**Figure S5.** Post-processing image analysis of laurdan in RBCs (see methods for further details)

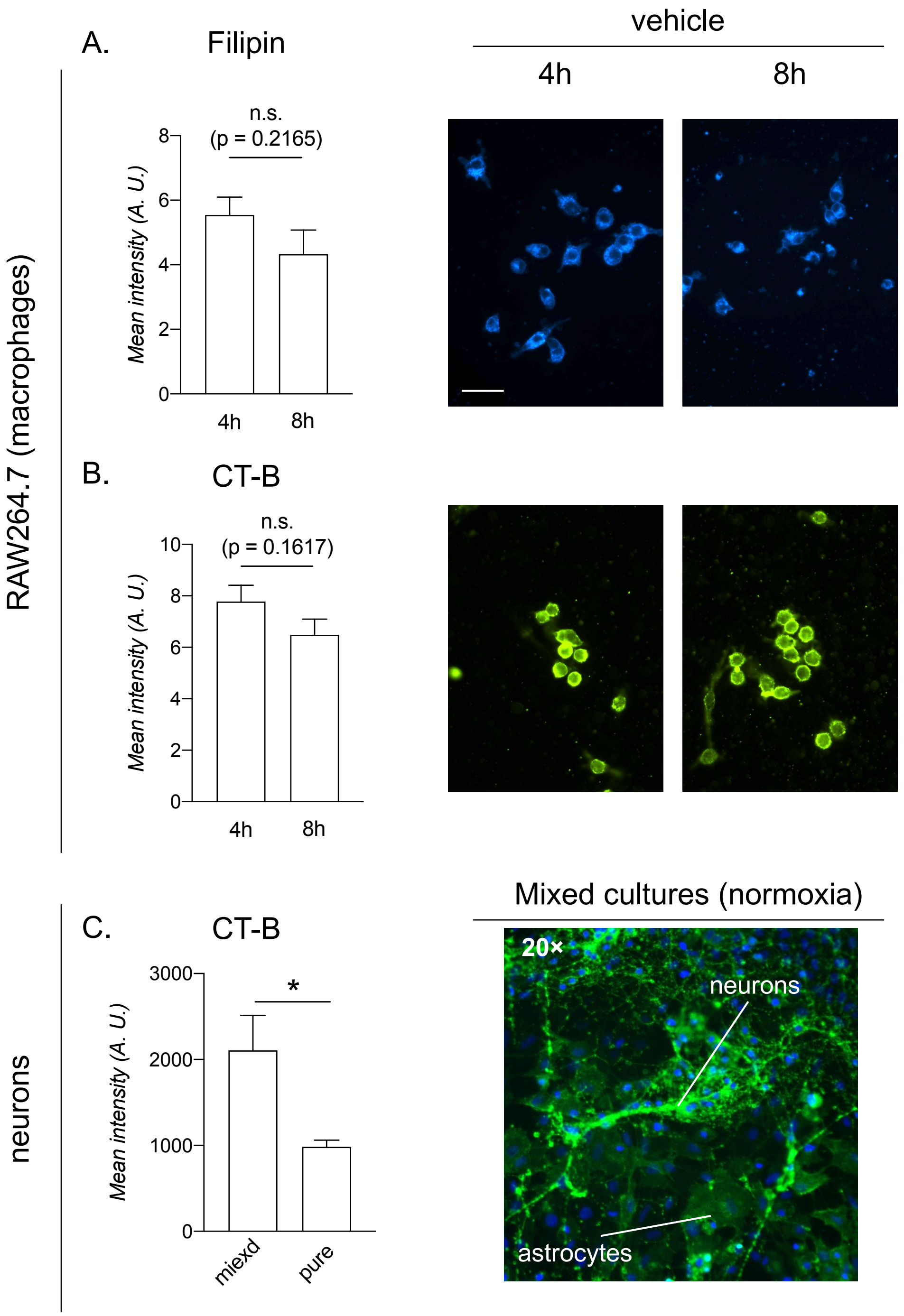


**Figure S6. Filipin and CT-B staining in control cultures.**

A-B. Graphs show the relative intensity of Filipin (A) and GM1 (B) represented as arbitrary units (A.U.) in non-treated cultures of macrophages cells (RAW264.7). Bars represent mean value + SEM. Statistical analysis: Student's t-test, p values are included in the graphs. Representative images acquired by epifluorescence microscope are included. Scale bar: 50 µm.

C. Graph shows the relative intensity of CT-B in neurons, represented as arbitrary units (A.U.), in mixed neuron/astrocyte (mixed) or pure neuronal (pure) cultures. Bars represent mean value + SEM. Statistical analysis: Student's t-test; asterisk represents differences between the two groups (* $p < 0.05$). A representative image, acquired by cell-based High Content Screening (20× objective) is included, containing the identification of neurons and astrocytes in control mixed cultures, based on the different CT-B staining.
